# Five Ways Speech-Language Pathologists Can Positively Impact Children With Dyslexia

**DOI:** 10.1044/2018_LSHSS-DYSLC-18-0102

**Published:** 2018-10-24

**Authors:** Tiffany P. Hogan

**Affiliations:** a MGH Institute of Health Professions, Boston, MA

## Abstract

The purpose of this epilogue is to offer five ways speech-language pathologists can positively impact children with dyslexia, drawing from and expanding on the articles presented in this clinical forum on dyslexia.

I have the privilege of working with and studying children and adults with dyslexia and using that knowledge to provide trainings for students and educators. A common theme is speech-language pathologists' lack of knowledge about dyslexia. It is my hope that reading the collection of articles presented in this clinical forum on dyslexia, written with the speech-language pathologist in mind, increases your knowledge base. Even with newfound (or confirmed) information about dyslexia, speech-language pathologists frequently have to contend with a lack of time, resources, and administrative support to implement what they've learned. In this epilogue to the forum, I offer five ways in which I believe speech-language pathologists can positively impact children with dyslexia despite these barriers.

## 1. Have Confidence in Your Expertise and Embrace the Role You Have in the Lives of Children With Dyslexia (Whatever That Role May Be)

It has been nearly two decades since the American Speech-Language-Hearing Association (ASHA, 2001) released its position statement on the roles and responsibilities of speech-language pathologists in reading. Since that time, countless publications have discussed, debated, and asserted our place in the assessment and intervention of reading disabilities (Catts & Kamhi, 2004; Ehren & Ehren, 2001; Kamhi, Allen, & Catts, 2001; Nelson, 2010; Schuele, 2017; Schuele & Larrivee, 2004; Spracher, 2000); however, based on a national sample of over 600 school-based speech-language pathologists, only 50% felt prepared to help struggling readers, and, further, 50% believed that literacy should not be in the scope of our practice (Katz, Maag, Fallon, Blenkarn, & Smith, 2010). The amount of time and coursework offered on reading in speech-language pathologist training is variable across programs, and in a similar survey, a majority of school-based speech-language pathologists “described their educational and clinical training as ‘limited’ in the evaluation and treatment of children with written language disorders” (Blood, Mamett, Gordon, & Blood, 2010, pg. 416). Moreover, it is not uncommon for me to hear from speech-language pathologists that, even though they feel confident in their ability to assess, treat, and consult on comprehension of written text, they do not feel the same confidence when assessing, treating, or consulting on word reading and dyslexia. In my view, it is the primary responsibility of clinical training programs to provide explicit instruction on ways in which speech-language pathologists contribute to the area of reading disabilities including dyslexia; to make clear that speech-language pathologists have critical phonological knowledge that can inform word-reading assessment and intervention; to educate speech-language pathologists on the process by which typical children develop sound–letter correspondence, orthographic knowledge, and fluent word reading; and to ensure that speech-language pathologists know that we can diagnose and treat dyslexia within our scope of practice.

Regardless of your past training, the fact that you are reading this epilogue shows your strong commitment to or curiosity about dyslexia, and you should be commended. Share informally and formally, far and wide, what you have learned about dyslexia. Collaborate with your peers to improve literacy practices in your schools. Start a multidisciplinary journal club to read articles about literacy acquisition, dyslexia, and evidence-based reading instruction. Feel confident in your language knowledge and your unique training in phonetic transcription and all things phonology. Partner with teachers, special educators, reading specialists, school psychologists, and administrators to improve literacy outcomes in your schools. Offer to discuss tough cases. It is in these big and small ways that you can positively impact children with dyslexia.

## 2. Seek Out Facts on Dyslexia and Share Those Facts With Your Colleagues and Parents

The compilation of articles in this forum provides a heap of factual information about dyslexia. Though dyslexia is a widely known condition, unfortunately it is also widely misunderstood. In fact, it is astounding how much misinformation there is about dyslexia (and learning disabilities in general). A recent survey found that over half of the surveyed public thought learning disabilities, including dyslexia, were the result of laziness and/or a poor home environment (Cortiella & Horowitz, 2014). Dispelling these harmful misconceptions by knowing fact from fiction is crucial to positively impacting children with dyslexia.

One rich and reliable source of factual information about dyslexia is the International Dyslexia Association (IDA, 2018) website, which includes fact sheets on many topics spanning from “dyslexia basics” to “effective reading instruction for students with dyslexia” and “applying for accommodations on college entrance exams.” The website also links to local IDA chapters; provides resources to help parents find professionals with specialized training to assess and treat children with dyslexia; contains printable infographics; and showcases success stories to inspire parents, educators, and those with dyslexia. Some key facts to share widely: Dyslexia is a lifelong condition (Saletta, 2018) that has a neurobiological basis (D'Mello & Gabrieli, 2018); children with dyslexia are found in all socioeconomic classes (from rich to poor; Shaywitz, 1996); children with dyslexia require intense, systematic, explicit word reading instruction (Al Otaiba, Rouse, & Baker, 2018); children with dyslexia often have co-morbid speech, language, and/or executive functioning impairments (Adlof & Hogan, 2018; Cabbage, Farquharson, Iuzzini-Seigel, Zuk, & Hogan, 2018); you do not need a brain scan to diagnosis dyslexia; and, contrary to popular press, dyslexia is NOT caused by visual problems such as seeing letters backwards (Vellutino, 1987).

## 3. Know how Dyslexia Relates to Other Communication Disorders

The hallmark of dyslexia is word reading below age- and grade-based expectations. Dyslexia is characterized as a “language-based” learning disability, because children with dyslexia have weak phonological processing (a component of the language system) that is presumed to be the primary cause of their poor word reading. Children with dyslexia often have early speech and language delays and later co-morbid deficits in broader language skills beyond phonology (Adlof & Hogan, 2018). This does not mean, however, that all children with dyslexia have language skills low enough to be classified as having developmental language disorder. In fact, children with dyslexia have a range of language skills from good to poor (Catts, Adlof, Hogan, & Ellis Weismer, 2005). Importantly, children with dyslexia who have “good” broader language skills often have subtle language deficits that likely reflect their weak phonological processing, such as difficulty learning to speak a foreign language and learning spoken labels for new words (Alt et al., 2017), and they are vulnerable to decreased language skills over time due to reduced opportunities to learn from texts (Cain & Oakhill, 2011). Because of these early and persistent speech and language deficits, speech-language pathologists are often the first professionals to assess and treat children who will go on to have dyslexia. In that role, speech-language pathologists can positively impact children with dyslexia by watching for the first signs of dyslexia, such as difficulty learning letters and letter-to-sound correspondences, and informing parents and educators about the increased risk of dyslexia in children with speech and language impairments as well as the increased risk of decreasing language skills over time in children with dyslexia.

## 4. Promote the Informed, Responsible, and Appropriate Use of the Dyslexia Label

Recent grassroots movements have resulted in almost all states passing legislation to “say dyslexia” in public schools (Ward-Lonergan & Duthie, 2018). With these changes to state laws, the dyslexia label is likely to be used more frequently in school settings. Though there have been debates on the pros and cons of labeling children as having dyslexia (Elliot & Grigorenko, 2014; Moats, 2008), according to these grassroots efforts, parents clearly believe the benefits of obtaining the label far outweigh potential risks. Those benefits include better identification and treatment of the word-reading deficits that characterize dyslexia and specialized dyslexia training for all educators. Of course, at this time, schools rarely use the dyslexia label, so children with dyslexia are likely unidentified; unlabeled in the Tier 2 system; or identified as developmentally delayed, specific reading disabled, and/or speech and language impaired.

Acknowledging the benefits of using a label, there are two points for consideration when diagnosing dyslexia. First, early diagnosis of dyslexia in pre-kindergarten or kindergarten is ideal so that early intervention may stave off the effects of future poor reading (Colenbrander, Ricketts, & Breadmore, 2018). Those effects include negative emotions associated with reading instruction, low self-esteem, and poor long-term reading outcomes. At present, early diagnosis is based on poor performance on tests of pre-reading skills such letter identification and phonological awareness. However, these tests only approximate a child's future reading ability and, as such, they can lead to over- and underidentification of dyslexia. Additionally, an increasingly diverse and multilingual school population further increases the possibility of over and underidentification of dyslexia (Yamasaki & Luk, 2018; but, see Petersen, Gragg, & Spencer, 2018). Considering the potential for misdiagnosis, those who fail pre-reading measures in pre-kindergarten or kindergarten are only *at risk* for dyslexia.

Another consideration when applying the label of dyslexia in schools is label stigma. Rappolt-Schlichtmann, Boucher, and Evans (2018) contrast the deficit model of dyslexia with neurodiversity, in which dyslexia is but one way individuals can differ from each other. Neurodiversity redirects disability away from a focus solely on deficits to including consideration of a child's strengths. Couching the label of dyslexia in this model can enable action towards remediation, positive prognosis, and access to information that may reduce the negative stigma that parents and children may experience from the dyslexia label. Promoting the informed, appropriate, and responsible use of the dyslexia label is a way speech-language pathologists can positively impact children with dyslexia.

## 5. Support Effective Interventions

Interventions that work to improve the reading skills of children with dyslexia also work to improve the reading skills of all children. These curricula involve systematic, explicit phonics instruction (see Al Otaiba et al., 2018). However, beware of wolves in sheep's clothing, that is, so-called phonics instruction that involves “discovery” or “balanced” literacy approaches. These approaches advertise evidence-based word reading instruction but instead may be based on principles of “whole language,” which has no scientific evidence (Castles, Rastle, & Nation, 2018). Children with dyslexia are particularly vulnerable to these watered-down approaches to word reading instruction, because they cannot simply “infer” sound–symbol correspondences and letter–sound patterns.

Speech-language pathologists may be particularly vulnerable to whole-language propaganda because its primary tenet is that strong language skills underpin reading. This fact is true, but reading has two components—word reading and comprehension—as illustrated by the simple view of reading (Hoover & Gough, 1990). Broad language skills—vocabulary, grammar, and higher-level discourse processing—underpin the comprehension component (Hogan, Bridges, Justice, & Cain, 2011), but phonology is the primary driver of learning to read words. Decades of research on the science of reading instruction show that word-reading skills are attained through explicit, systematic phonics instruction for almost all children. Likewise, explicit, systematic, and clear writing instruction is critical for children with dyslexia, as discussed by Hebert, Kearns, Hayes, Bazis, and Cooper (2018). Because children with dyslexia often have co-morbid language impairments, it is imperative that these children receive not only strong word-reading interventions but also language interventions to facilitate growth in broader speech and language abilities in order to ensure good text comprehension.

Many resources are available to determine if an intervention has strong evidence to support its assertion of effectiveness. One such resource is the Academic Interventions tools chart from the National Center on Intensive Intervention at American Institutes for Research (n.d.), which provides a consumer reports–type rating of the evidence base for numerous popular reading interventions. There are also study details and implementation information. Additionally, the What Works Clearinghouse (n.d.) provides intervention reports, practice guides, and individual study reviews to help determine the effectiveness of specific curricula. As an informed speech-language pathologist, you can use this information to help guide intervention decisions in your schools and to discuss the pros and cons of different interventions. Finding, promoting, and supporting effective reading, language, and writing interventions is a way speech-language pathologists can positively impact children with dyslexia.

## Conclusion

When I first began practicing speech-language pathology, the notion of a clinical forum in an ASHA journal devoted to the topic of dyslexia seemed far-fetched (though there were pioneering speech-language pathologists contributing to clinical practice and research on dyslexia). A few decades later, it is my privilege to lead the first clinical forum on this important topic. I am hopeful that the information presented will help current and future speech-language pathologists go forth confidently to positively impact children with dyslexia.
